# SARS-CoV-2 & Rheuma: Konsequenzen der SARS-CoV-2-Pandemie für Patienten mit entzündlich rheumatischen Erkrankungen. Ein Vergleich der Handlungsempfehlungen rheumatologischer Fachgesellschaften und Risikobewertung verschiedener antirheumatischer Therapien

**DOI:** 10.1007/s00393-020-00878-0

**Published:** 2020-08-26

**Authors:** J. Leipe, B. F. Hoyer, C. Iking-Konert, H. Schulze-Koops, C. Specker, K. Krüger

**Affiliations:** 1 Sektion Rheumatologie, Medizinische Klinik V, Universitätsklinikum Mannheim, Theodor-Kutzer-Ufer 1–3, 68167 Mannheim, Deutschland; 2 Sektion Rheumatologie und Klinische Immunologie, Medizinische Klinik und Poliklinik IV, Ludwig-Maximilians-Universität München, München, Deutschland; 3 Abteilung für Rheumatologie, 1. Medizinische Klinik, Universitätskrankenhaus Schleswig-Holstein Campus Kiel, Kiel, Deutschland; 4 III. Medizinische Klinik und Poliklinik Sektion Rheumatologie, Universitätsklinikum Hamburg-Eppendorf, Hamburg, Deutschland; 5 Klinik für Rheumatologie und Klinische Immunologie, Kliniken Essen-Mitte, Essen, Deutschland; 6 Rheumatologisches Praxiszentrum München, München, Deutschland

**Keywords:** COVID-19, Management, Empfehlungen, Entzündlich rheumatische Erkrankungen, DMARD, COVID-19, Management, Recommendations, Inflammatory rheumatic diseases, DMARD

## Abstract

Die Empfehlungen des Deutschen Gesellschaft für Rheumatologie(DGRh)-Updates – welches die zu Beginn der COVID-19-Pandemie erstellte Hilfestellung zum Management von Patienten mit entzündlich rheumatischen Erkrankungen angesichts der Bedrohung durch SARS-CoV‑2 aktualisiert und erweitert – stimmen in vielen Punkten mit den Handlungsempfehlungen der amerikanischen (ACR) und europäischen Fachgesellschaften (EULAR) überein, unterscheiden sich aber auch in einigen Punkten. In diesem Artikel sollen daher Kernempfehlungen des DGRh-Updates zu den Themen Prävention von SARS-CoV-2/COVID-19, Risikoeinschätzung bei ERE sowie der Umgang mit antirheumatischen Therapien im Kontext bzw. im Vergleich zu den ACR- und EULAR-Empfehlungen diskutiert und eine Übersicht zur Risikobeurteilung einzelner antirheumatischer Medikamente gegeben werden.

## Abstract (en)

The recommendations of the German Society of Rheumatology (DGRh) update, which update and expand the guidance on the management of patients with inflammatory rheumatic diseases in view of SARS-CoV‑2 created at the beginning of the COVID-19 pandemic, correspond in many points with the recommendations for action of the American (ACR) and European (EULAR) societies, but also differ in some points. Therefore, this article discusses the core recommendations of the DGRh update on the prevention of SARS-CoV-2/COVID-19, the risk assessment for inflammatory rheumatic diseases and the use of antirheumatic treatments in the context and in comparison to the ACR and EULAR recommendations, and provides an overview of the risk assessment of individual antirheumatic drugs.

Nachdem die Deutsche Gesellschaft für Rheumatologie e. V. (DGRh) bereits zu Beginn der COVID-19-Pandemie im März 2020 erste Handlungsempfehlungen zum Management von Patienten mit entzündlich rheumatischen Erkrankungen (ERE) unter dem Aspekt der SARS-CoV-2/COVID-19-Bedrohung herausgegeben hat [13], wurden diese im Juli 2020 durch ein Update aktualisiert und erweitert (im Folgenden *DGRh-Update*) [14]. Während die initialen Empfehlungen in Ermangelung von Evidenz nahezu ausschließlich auf Expertenkonsens basierten, beruht das DGRh-Update auf der Datengrundlage von Registern, Querschnittstudien und Fallserien, welche bereits in kurzer Zeit wichtige Informationen zu SARS-CoV-2/COVID-19 bei ERE lieferten. Sämtliche dieser Datenquellen weisen Limitationen auf: Den wichtigsten potenziellen Bias stellt dabei u. a. die für diese Studienformen charakteristische überproportionale Erfassung schwerer Fälle dar; zudem auch das Fehlen von parallelen, regionalen Vergleichskohorten ohne ERE genauso wie der regional unterschiedliche Umgang mit SARS-CoV‑2 bzw. die unterschiedliche Häufigkeit sowie die spezifischen Unterschiede der einzelnen Gesundheitssysteme.

Im DGRh-Update wurden Aspekte der Prävention bzw. des Managements von SARS-CoV-2/COVID-19, eine Risikoeinschätzung bei ERE sowie der Umgang mit immunmodulatorischen/immunsuppressiven Medikamenten bei diesen Patienten thematisiert. Die Empfehlungen stimmen in vielen Punkten mit denen der amerikanischen (*American College of Rheumatology* [ACR]) [10] und europäischen (*European League Against Rheumatism* [EULAR]) [8] Fachgesellschaften überein, unterscheiden sich aber auch in einigen Punkten. Im Folgenden sollen daher Kernempfehlungen des DGRh-Updates im Kontext bzw. im Vergleich zu den ACR- und EULAR-Empfehlungen näher beleuchtet und eine Übersicht zur Risikoeinschätzung einzelner antirheumatischer Medikamente gegeben werden.

## Prävention/Management von SARS-CoV-2/COVID-19

Alle 3 Fachgesellschaften empfehlen die Einhaltung der seitens lokaler Behörden verordneten Verhaltens- und Vorsichtsmaßnahmen analog zur Allgemeinbevölkerung. Darüber hinausgehende Maßnahmen (z. B. der Gebrauch von FFP2/3-Masken für besonders gefährdete Patienten) werden mangels wissenschaftlicher Evidenz nicht empfohlen. Unterschiede bestehen bezüglich Arztkontakten aufgrund der rheumatischen Erkrankung: Während ACR und EULAR bei kontrollierter Erkrankung und stabiler Therapie ausgewählte Maßnahmen zur Verringerung von persönlichen Kontakten – z. B. weniger Laborkontrollen, Nutzung von Telemedizin, verlängerte Dosierungsintervalle bei i.v.-Medikamenten und Verschiebung von rheumatologischen Vorstellungen – vorschlagen, wird von der DGRh eine normale Versorgung der Patienten mit ERE empfohlen. Dieser Unterschied ist sicher durch die momentan besser kontrollierte Infektionssituation in Deutschland gegenüber anderen europäischen Ländern und den USA zu erklären. Darüber hinaus zeigen neuere Veröffentlichungen, welche zum Zeitpunkt der Erstellung der ersten DGRh- sowie der ACR- und EULAR-Empfehlungen größtenteils noch nicht vorlagen, dass kein generell erhöhtes Risiko für Patienten mit ERE besteht [1, 5–7, 9, 11]. Basierend darauf, überwiegt nun die Sorge vor einem potenziellen Schaden infolge (ungerechtfertigter) Einschränkungen der Versorgung („medizinischer Kollateralschaden“) gegenüber einem Infektionsrisiko durch Arztbesuche. Allerdings ist die Rückkehr zur normalen Versorgung in Deutschland auch an „Bedingungen“ geknüpft. So sollen Praxen und Ambulanzen entsprechende Verhaltens- und Hygienemaßnahmen sowie eine intelligente Sprechstundenplanung gewährleisten mit u. a. kürzeren Wartezeiten (weniger Patienten gleichzeitig in den Praxen und Ambulanzen), Einhalten nötiger Abstandsregeln und die maximale Reduktion der Zahl von Begleitpersonen, um Risiken zu minimieren. Zudem sollen die Patienten möglichst im Vorfeld systematisch abgefragt bzw. informiert werden, nicht mit Krankheitssymptomen von COVID-19, nicht bei Rückkehr aus Risikogebieten (innerhalb der letzten 14 Tage; auch nicht nach initial negativem Testergebnis [16]) oder nach Kontakt zu nachweislich SARS-CoV-2-Infizierten in die Einrichtung zu kommen. Dies gilt auch für möglicherweise unabdingbare Begleitpersonen. Ausweichstrategien für Patienten mit Infusionsterminen in diesem Quarantänezeitraum sollten erarbeitet werden (ggf. stationäre Aufnahmen). In Hochrisikogebieten können striktere Verhaltens- und Vorsichtsmaßnahmen gelten.

## Risikoeinschätzung für Patienten mit entzündlich-rheumatischen Erkrankungen

Bezüglich der Risikoeinschätzung für schwere COVID-19-Verläufe wurde in den Empfehlungen der DGRh, EULAR und ACR postuliert, dass Patienten mit ERE kein grundsätzlich erhöhtes Risiko einer Infektion mit SARS-CoV‑2 oder eines schweren Verlaufes für COVID-19 aufweisen. Vielmehr wurde in allen 3 Empfehlungen davon ausgegangen, dass das Risiko für einen schweren Verlauf in erster Linie mit den allgemeinen COVID-19-Risikofaktoren wie Alter und Komorbiditäten zusammenhängt [2]. Die deutsche Empfehlung geht, u. a. basierend auf teilweise kurz davor publizierten Daten, davon aus, dass auch die medikamentöse antirheumatische Therapie kein Risiko für einen schweren Verlauf von COVID-19 bei Patienten mit ERE darstellt, mit Ausnahme von Glukokortikoiden in einer Dosierung von 10 mg Prednisolonäquivalent/Tag und mehr [4]. Die evidenzbasierte Risikoeinschätzung steht damit im Widerspruch zu der Bewertung des RKI, welche generell von einem erhöhten Risiko bei Einnahme von immunmodulatorischen Medikamenten ausgeht [17]. Bei der Einschätzung des RKI, die sich bereits seit Beginn der COVID-19-Krise auf der Webseite findet, handelt es sich vermutlich um eine Annahme; zudem ist sie, was die Bewertung unterschiedlicher Formen der Immunsuppression angeht, unpräzise. Unseres Erachtens sollte diskutiert werden, diese Angabe zu aktualisieren. Es ist jedoch nicht auszuschließen, dass zukünftige Daten noch (über das bei >10 mg Prednisolonäquivalent/Tag hinausgehende) weitere medikamentöse Risikofaktoren ergeben. Aufgrund der derzeit vorliegenden Daten erscheint es unwahrscheinlich, dass dies auf alle ERE-Patienten zutrifft. Einzelne Subgruppen könnten allerdings doch ein erhöhtes Risiko für schwere Verläufe von COVID-19 haben. Es scheint aktuell plausibel, dass das insgesamt niedrige Risiko unserer Patienten auch (und v. a.) an deren Umsichtigkeit im Umgang mit den empfohlenen Präventionsmaßnahmen liegt. Die Patienten sollten deshalb unbedingt angehalten werden, diese weiter konsequent durchzuführen.

Im DGRh-Update wurde, basierend auf aktuell publizierten Daten zu SARS-CoV‑2 und Erkenntnissen aus früheren Studien zum allgemeinen Infektionsrisiko, von einem erhöhten Risiko für einen schweren COVID-19-Verlauf bei unzureichend eingestellten ERE ausgegangen [3]. Basierend auf diesen Erkenntnissen, sollte daher bei Patienten mit gut eingestellter Erkrankung die antirheumatische Therapie nicht aus Sorge vor Komplikationen mit SARS-CoV‑2 abweichend vom üblichen Vorgehen verändert oder pausiert werden. Jede Veränderung der laufenden Therapie bedeutet eine potenzielle Destabilisierung der Krankheitskontrolle.

## Umgang mit immunmodulatorischen/immunsuppressiven Medikamenten

### Antirheumatische Therapie bei Patienten ohne Infektzeichen

Das DGRh-Update unterscheidet zwischen bestehender und Neubeginn/Umstellung einer antirheumatischen Therapie. Bei bestehender Therapie sollen nichtsteroidale Antirheumatika (NSAR), Glukokortikoide (GC, <10 mg/Tag Prednisolonäquivalent), „*conventional synthetic disease modifying anti-rheumatic drugs“* (csDMARDs), „*targeted synthetic* DMARDs“ (tsDMARD), „biological DMARDs“ (bDMARDs) und Immunsuppressiva unverändert fortgesetzt werden. Ähnlich empfiehlt auch die EULAR, NSAR, GC und DMARDs unverändert zu belassen, und die ACR-Taskforce, NSAR, GC (≤10 mg/Tag Prednisolonäquivalent), DMARDs und Immunsuppressiva fortzusetzen.

Bei notwendigem Neubeginn/Umstellung einer antirheumatischen Therapie sollte gemäß DGRh-Empfehlung eine Behandlung nicht aufgrund der COVID-19-Pandemie unterbleiben, verändert, verzögert oder unterdosiert werden. Generell sollte nach guter klinischer Praxis gemäß den aktuell geltenden Leitlinien für die entsprechenden Krankheitsbilder behandelt werden. Bei bestehenden Alternativen kann erwogen werden, eher Substanzen mit kurzer Halbwertszeit zum Einsatz zu bringen. Eine Präferenz für eine besondere Substanz oder Substanzklasse sprechen die Empfehlungen nicht aus. Es sollte aber – wie bisher – auf den unnötigen Einsatz von hoch dosierten Glukokortikoiden verzichtet werden.

Die ACR-Taskforce unterscheidet bezüglich des Neubeginns der Therapie noch zwischen Patienten mit SLE, Arthritiden und anderen ERE, obgleich angemerkt werden muss, dass die Empfehlungen zu diesen spezifischen Situationen noch mehrheitlich Expertenmeinungen darstellten und zu dem damaligen Zeitpunkt wenig evidenzbasiert waren. Demnach sollen Antimalariamittel unverändert bei neu diagnostiziertem systemischem Lupus erythematodes (SLE) in voller Dosis begonnen und bei Schwangeren mit gleicher Dosis fortgeführt werden; wenn indiziert, kann Belimumab initiiert werden. Patienten mit Arthritis sollen gemäß ACR, sofern gut kontrolliert, unverändert mit Antimalariamitteln und IL-6R-Inhibitoren (IL-6Ri) weiterbehandelt werden. Bei aktiver oder neu diagnostizierter Arthritis können csDMARDs begonnen oder umgestellt werden. Bei moderater bis hoher Krankheitsaktivität trotz csDMARD-Therapie können Biologika gestartet werden.

Speziell zu GC empfehlen DGRh und ACR, diese in der niedrigstmöglichen Dosis zur Kontrolle rheumatischer Erkrankungen einzusetzen, möglichst ≤10 mg Prednisolonäquivalent/Tag. Eine GC-Dauertherapie <10 mg/Tag Prednisolonäquivalent sollte in gleicher Dosis fortgesetzt und moderat hoch dosierte und/oder niedrig dosierte Glukokortikoide sollten nicht abrupt abgesetzt werden, unabhängig von der SARS-CoV-2-Exposition oder dem Infektionsstatus. Zudem können selbst nach SARS-CoV-2-Exposition hoch dosierte Glukokortikoide im Kontext schwerer, vital organbedrohlicher Manifestation der rheumatischen Erkrankung (z. B. Lupusnephritis oder Vaskulitis) notwendig sein.

### Antirheumatische Therapie bei Patienten mit Kontakt zu SARS-CoV-2-positiven Individuen und ohne eigene COVID-19-Infektzeichen

Hier empfiehlt die DGRh zunächst eine Fortführung der Therapie und die Kontaktaufnahme mit dem Rheumatologen bei Auftreten von Symptomen. Das ACR empfiehlt dagegen bereits in dieser Konstellation das Pausieren von Immunsuppressiva, nicht aber von IL-6Ri-Biologika und JAK-Inhibitoren (JAKi), temporär bis zum Vorliegen eines negativen COVID-19-Tests oder nach 2 Wochen symptomfreiem Beobachtungszeitraum. Unter bestimmten Umständen, z. B. bei Riesenzellarteriitis, können gemäß ACR IL-6Ri fortgeführt werden. Die Empfehlung, IL6Ri und JAKi nicht abzusetzen, beruht auf der Annahme, dass eine immunmodulierende Therapie einen sog. „Zytokin-Sturm“ und dadurch schwerste Verläufe von COVID-19 ggf. verhindern kann. Insbesondere für Tocilizumab gab es schon früh Einzelberichte und Fallserien, dass sich u. a. die Notwendigkeit und Dauer von Beatmungspflichtigkeit, aber auch die Mortalität möglicherweise senken lässt [2]. Die nun kürzlich zunächst in Pressemitteilungen veröffentlichen ersten Informationen zur Phase-III-Studie von Tocilizumab und Sarilumab konnten diese Erwartung aber bisher nicht erfüllen [18, 19]; weitere Phase-III-Studien mit Tocilizumab und auch Sarilumab befinden sich aber noch in Auswertung.

Die EULAR-Empfehlungen nehmen zu dieser Konstellation keine Stellung.

### Antirheumatische Therapie bei Patienten mit Kontakt zu SARS-CoV-2-positiven Individuen und mit eigenen COVID-19-Infektzeichen

Hier empfiehlt die DGRh bei leichten Symptomen und fehlendem Fieber keine Therapieänderung, aber eine Kontaktaufnahme mit dem Rheumatologen und bei eindeutigen Infektzeichen, insbesondere Fieber (>38 °C), eine Pausierung der antirheumatischen Medikation. Zu dieser speziellen Konstellation nehmen weder EULAR noch ACR dezidiert Stellung.

### Antirheumatische Therapie bei positiv auf SARS-CoV-2 getesteten (PCR) Patienten ohne COVID-19-Infektzeichen

Für diese Konstellation empfiehlt die DGRh ein Pausieren oder Herauszögern einer ts- oder bDMARD-Therapie für die Dauer der mittleren Inkubationszeit. Bei Symptomfreiheit sollte noch eine Pause für 5 bis 6 Tage nach Abstrich erwogen werden; csDMARDs sollten nicht abgesetzt werden.

### Antirheumatische Therapie bei positiv auf SARS-CoV-2 getesteten (PCR) Patienten mit COVID-19-Infektzeichen

Für diese Konstellation einer COVID-19-Erkrankung empfiehlt die DGRh ein Pausieren der DMARD-Therapie. Bei Einnahme von Leflunomid sollte wegen der langen Halbwertszeit ein Auswaschen erwogen werden, wobei dies angesichts fehlender Sicherheitssignale in COVID-19-Registern und möglicher (derzeit in Studien getesteter) positiver Wirkung des Metaboliten Teriflunomid bei COVID-19 durchaus diskussionswürdig ist.

Bei dokumentierter oder vermuteter COVID-19-Erkrankung empfiehlt das ACR in ähnlicher Weise Sulfasalazin, Methotrexat, Leflunomid, Immunsuppressiva, Nicht-IL-6Ri-Biologika und JAKi zu pausieren (Begründung s. oben), jedoch Antimalariamittel fortzusetzen. Letzteres wurde mangels überzeugender Daten zur zunächst erhofften positiven Wirkung von Hydroxychloroquin (HCQ) auf den Verlauf von COVID-19 seitens der DGRh nicht explizit empfohlen.

## Risiko antirheumatischer Therapie bei COVID-19

Eine Übersicht über die momentane Einschätzung zu den einzelnen Substanzen/Substanzklassen ist in Tab. 1 zusammengefasst. Diese Einschätzung basiert auf der Literatursuche, die im Rahmen der Erstellung des DGRh-Updates durchgeführt wurde.

**Table 1:** 

Substanzklassen/Substanzen	Effekt auf COVID-19-Verlauf	Sonstige Bemerkungen
*NSAR*	⇒	Initial diskutiertes Risiko nicht belegt
*csDMARD*		
Hydroxychloroquin	⇒	Initial diskutierter protektiver Effekt aktuell nicht belegt (Zitat u. a. Cochrane)
Sulfasalazin	⇒	–
Methotrexat	⇒	–
Leflunomid	⇒	–
Mycophenolat-Mofetil/Azathioprin/Calcineurininhibitoren	Ø	–
*bDMARD*		
Abatacept	⇒	Potenziell eingeschränkte Impfantwort bei COVID-19-Impfung?
Belimumab	⇒	–
IL-1-Inhibitoren	⇒	Protektive Wirkung bei sehr schweren Verläufen beschrieben RCTs zum Einsatz bei COVID-19 laufen
IL-6R-Inhibitoren	⇒	Protektive Wirkung bei sehr schweren Verläufen in Fallserien Zahlreiche RCTs zum Einsatz bei COVID-19 laufen. Die ersten beiden berichteten Phase-III-Studien (NCT04320615, NCT04315298) ohne signifikanten Effekt
IL-12/23-Inhibitoren	⇒	–
IL-17-Inhibitoren	⇒	–
Rituximab	↗⇗	Einzelfallberichte mit kritischem Outcome. Potenziell eingeschränkte Impfantwort bei COVID-19-Impfung?
TNF-Inhibitoren	⇒	Protektive Wirkung diskutiert RCTs zum Einsatz bei COVID-19 laufen
*tsDMARD*		
Apremilast	⇒	–
JAK-Inhibitoren	⇒	RCTs zum Einsatz bei schwerem COVID-19 laufen

Risiko für schweren Verlauf von COVID-19 aus wissenschaftlichen Daten: keine Hinweise für erhöhtes Risiko: ↗, einzelne Fälle von schweren Verläufen: ⇒, keine Daten oder nur wenige Fallberichte: Ø

*aOR* adjustierte Odds Ratio, *bDMARD* „biologic disease modifying anti-rheumatic drugs“ (bDMARDs), *csDMARDs* „conventional synthetic DMARDs“, *RCTs* „randomized controlled trials“, *tsDMARD* „targeted synthetic DMARDs“

Ob sich möglicherweise sogar günstige Effekte von Glukokortikoiden, bDMARDs und tsDMARDs auf den Verlauf von COVID-19 bei Patienten mit ERE und/oder ohne ERE aus randomisiert kontrollierten Studien ergeben, wird sich zukünftig zeigen. Wie oben diskutiert, haben sich die zunächst hoffnungsvollen Daten u. a. zu HCQ und IL-6Ri in weiteren Studien nicht erfüllt, während es für eine Intervention mit Glukokortikoiden positive Signale gibt [15].

## Fazit für die Praxis

Die vorgestellten Empfehlungen, v. a. das DGRh-Update, kommen nach Bewertung der vorliegenden wissenschaftlichen Daten zu der vorläufigen Schlussfolgerung, dass für Patienten mit ERE kein generell erhöhtes Risiko für die Häufigkeit oder einen schweren Verlauf einer COVID-19-Erkrankung besteht, es sei denn, es liegen der allgemeinen Bevölkerung entsprechende Risikobegleiterkrankungen, eine erhöhte Krankheitsaktivität oder eine Glukokortikoideinnahme von mehr als 10 mg Prednisolonäquivalent/Tag vor.

Auf der Basis dieser Daten empfiehlt die DGRh im Vergleich zu den Empfehlungen der EULAR und des ACR eine weniger restriktive Haltung bezüglich einer Vermeidung von Arztkontakten. Basistherapien sollten – im Gegensatz zu EULAR und ACR – nur beim Vorliegen eindeutiger Infektsymptome oder einem positiven SARS-CoV-2-Nachweis pausiert werden.

Bezüglich der Bewertung der einzelnen Substanzen ist an dieser Stelle noch einmal zu betonen, dass die Datenbasis für die Empfehlungen aus Fallserien, Kohortenstudien und Fall-Kontroll-Studien besteht und der Evidenzgrad somit noch begrenzt ist. Dies und Confounder, die nur begrenzt etwas mit der Substanz oder der rheumatischen Grunderkrankung zu tun haben, erschweren eine endgültige Bewertung des Risikos für schwere COVID-19-Verläufe. So werden bestimmte Substanzen vorrangig bei Patienten mit bestimmtem Vor‑/Begleiterkrankungen, wie z. B. Malignomen oder Herzinsuffizienz, eingesetzt, was hier zu einer Verzerrung führen kann. Auch können wir nicht abschließend beurteilen, inwiefern die aktuellen Daten zu Häufigkeit und Schwere von COVID-19 bei Patienten mit ERE durch ein besonders vorsichtiges Verhalten und eine restriktive Umsetzung der Präventionsmaßnahmen vonseiten unserer Patienten gegenüber der Normalbevölkerung begründet ist.

Diese Faktoren limitieren zwar die Qualität aller Empfehlungen, trotzdem kann an dieser Stelle zumindest ein vorsichtiges Signal der Entwarnung gegenüber der ganz initialen Sorge um unsere Patienten gegeben werden, dass sie per se oder durch die spezifische Therapie einem höheren Risiko für eine SARS-CoV-2-Infektion oder einem schweren Verlauf einer COVID-19-Erkrankungen ausgesetzt seien. Zukünftig ist zu erwarten, dass DGRh, ACR und EULAR, basierend auf neuen wissenschaftlichen Daten (auch eigener klinischer Register), weitere konkretere Handlungsempfehlungen veröffentlichen.
